# Monthly Summary

**Published:** 1868-07

**Authors:** 


### MONTHLY SUMMARY.

*The New Anæsthetic (?) Nitrous Oxide.*—A very opportune discussion took place at the Medical Society of London, on Monday night last, on the so called Anæsthetic Nitrous Oxide Gas. A question on the subject addressed to the President, Dr. Richardson, whose authority on such a point cannot be questioned, drew from him a clear and careful summary of its action. It was painful, he remarked, to see the childish excitement with nitrous oxide and its effects had recently been dwelt on. The gas had been treated as an unknown, wonderful, and perfectly harmless agent; whereas, in simple fact, it was one of the best known, least wonderful, and most dangerous of all the substances that had been applied for the production of general anæsthesia. No substance had been physiologically studied with greater scientific zeal or more rigid accuracy; and no substance had been more deservedly given up as unfit and unsafe for use. It had caused death in the human subject, and on animals it was so fatal that with the utmost delicacy in its use, it was a critical task thoroughly to narcotize an animal with the gas without actually destroying life. In some cases, also, animals died after recovering from the insensibility

Respecting the modes of action of the nitrous oxide, Dr Richardson explained that it was not, in the true sense, the agent that caused the insensibility. It acted indirectly, and the immediate stupefier was really carbonic acid. In fact, nitrous oxide is an asphyxiating agent. There are two explanations of this. It may be that the nitrous oxide quickens the oxidation of blood, and so causes accumulation of carbonic acid in the blood; or it may be—and this is most probable—that it acts by checking the outward diffusion of carbonic acid. The vapor density of nitrous oxide and of carbonic acid is the same—namely, 22, taking hydrogen as unity; and as diffusion of gases into the blood and out of it, is governed by the same laws as in ordinary diffusion, to make an animal breathe nitrous oxide is virtually equivalent to making it breathe carbonic acid itself, the diffusion of carbonic being so determinately impeded. The living phenomena were also in character; the arterial blood was rendered venous by nitrous oxide; the animal temperature fell; the skin became livid. And although these symptoms might be induced many times without actually destroying life, they could not be sustained for any length of time without certain disaster. Dr. Sansom followed in nearly the same strain

In speaking out thus boldly to a professional audience, Dr. Richardson has not spoken a moment too soon. The ad captandum method of applying the most potent medicinal agents against the teachings of scientific experiment and the experience of accepted observers, is a phrase in physic which requires to be put down with a strong hand. Administration of nitrous oxide, or laughing gas as it is commonly called, is becoming a pastime for amateurs. We hope these few and timely words will prevent a catastrophe. If they fail, the fault or neglect will not rest with us.—*Lancet*

---

*Poisoning by Carbolic Acid.*—In the current volume of the "Guy's Reports" a very interesting case of poisoning by carbolic acid is mentioned by Dr. A. S. Taylor. Having had a somewhat similar case occur in my practice from the same poison, and feeling its effects have as yet been but little tested, I trust the following brief facts may be acceptable to some of our readers

On the evening of March 18, I was hastily summoned to see S. C., aged 43, who had accidently taken oz. j of carbolic acid (which was kept in the wards for disinfecting purposes) instead of a dose of black draught. I saw her within five minutes after the poison was taken. She was reclining in a chair insensible ; her face was blanched and bathed in perspiration, pupils contracted ; pulse 100, feeble, and very intermittent: respiration stertorous and smelling strongly of the fluid ; there was slight lividity of the lips and tips of the fingers. She rapidly became worse, and died within an hour and a half of taking the poison, the body becoming much swollen a short time before death.

The only treatment employed was an emetic and the stomach-pump. The former she could not swallow, and the latter only brought back a small quantity of the contents of the stomach, which smelt strongly of the fluid. I found great difficulty in passing a tube down the œsophagus, owing to the spasmodic contraction of its walls.

The post-mortem examination was made seventeen hours after death G. W. W. Firth, Esq, Consulting Surgeon of the Asylum and Surgeon to the Norfolk and Norwich Hospital, and Dr. Beverley, House-Surgeon of the latter institution, kindly assisting me. Body well nourished. At the angles of the mouth the skin was rather discolored and shrivelled ; the interior of the mouth was very white ; tongue dry and chippy ; the mucous membrane of the œsophagus was dry and shrunken, and of a brownish color. The lungs were healthy. Heart flabby, and both the ventricles were empty ; the valves were healthy. The stomach contained about a pint and a half of food, consisting of partly digested meat, bread, etc. The mucous membrane could be readily peeled from the walls of the stomach. There were several peculiar dry white patches on the surface of the rugæ, and the whole of the interior of the stomach was slightly inflamed; the walls of the duodenum were similarly affected though in a much slighter degree. The liver was small and slightly lobulated. Spleen and kidneys healthy. Brain : The dura mater was natural, arachnoid thickened and opaque, the Pacchionian bodies unusually distinct. (I have generally noticed this in all the brains of the insane that I have examined.) The brain substance was apparently healthy.—*Dr. Sutton, in Medical Times and Gazette.*

---

*Local Anæsthesia in the Treatment of Traumatic Tetanus.*—J. C. Whitehill, M. D. reports having been called to a case of tetanus, in a boy of about 16 years, who had trodden upon a rusty nail. The wound, although temporarily painful, healed over in a few days, and gave no further trouble. In about ten weeks, he complained of slight stiffness of the muscles of the neck and a difficulty in swallowing, which increased until well-marked symptoms of tetanus were recognized. The usual routine of treatment was tried without any apparent benefit. The inhalation of chloroform was thoroughly resorted to, and afforded relief *only* when total anæsthesia was produced.

The application of chloroform to the entire spinal column was then made by means of a cloth saturated with it, and evaporation prevented by covering the cloth with oiled silk. The application was made just at the approach of a paroxysm. As a result of the application, the paroxysm was averted, and in a very few minutes the patient fell into a calm and natural sleep, lasting several hours —the longest interval between the paroxysms, he had yet enjoyed. On feeling a returning paroxysm, he asked for a re-application; and a second time the spasm was averted, and a comfortable sleep followed. For the next forty-eight hours occasional tetanic symptoms immediately yielded to the application of chloroform. The subsequent convalescence was rapid. Three cases reported by Dr. Hinkle gave a like favorable result from the same method of treatment.—*Humboldt Medical Archives.*

---

*The Cause of Caries in Teeth.*—It has been established by an English surgeon (Dr. I. P. H. Brown) that this condition of the teeth commences to manifest itself in many women after fecundation, for, during that state the mother is called on to furnish precisely this calcareous element, for the development of the fœtal structure ; and when this assimilating material is insufficient, the fœtal organization must be supplied at the cost of the bone proper of the teeth—a material difficult of repair, but admitting of ready and rapid deterioration. Dr. Mayitot. in his new treatise on dental caries, demonstrates that the saliva of the mouth, in consequence of the change in its composition, in various diseases, as typhoid fever, dyspepsia, etc., exercises a hurtful influence, and is a true cause of dental caries.—*GazetaMed. da Bahia.*

---

*A new Anœsthetic.* The indefatigable Dr. Richardson has introduced a new anæsthetic, methylic ether which is obtained from methylic alcohol, and pyoxylic spirit, by testing it with sulpheric acid and then purifying it. It is a gas but is soluble in ether and alcohol. One volume of water takes up thirty-seven volumes of the gas. Its formula is $(C\,H_3\,)_2\,O$ ($C=12$ and $O=16$) and the density of its vapor, 23. Blood absorbs more than half as much more of this than of ether vapor.

Dr. Richardson employs a solution of it in ether for anæsthetic purposes. By the solution, anæsthesia is rapidly and easily produced, less than a minute being the time required. It appears to be unattended with any of the ordinary discomforts. It is preceded by no agitation or convulsion and followed by no nausea. In an experiment on his own person, the Doctor found his pulse to rise to 96. He recommends it for rapid operations such, for example as the extraction of teeth.

---

*Death from Chloroform*—The following case is from Dr. B. F. Brown of Oneida Ill. published in the *Chicago Med. Examiner.*

Mrs. Elizabeth Hammon, aged 35, mother of three children, apparently a healthy woman, went to a dentist (Dr. McDowell) April 16, 1868 for the purpose of having some teeth extracted. Thinking she could not endure the pain, she requested the Doctor to administer chloroform; and, as he had given it to her once before (about six months since) without any bad effect, he consented to administer it again, and pouring about two drachms of chloroform upon a sponge, held it a short distance from her face. After she had made three or four inspirations her respiration ceased; he felt for the pulse and found she had none. Artificial respiration was commenced at once, and stimulating applications applied over the heart and to the extremities, but to no effect. She made but two or three efforts at inspiration after he first noticed something was wrong. An autopsy could not be obtained.

---

*Fatal Hemorrhage after Extraction of a Tooth.*—Dr. Schunemann relates an interesting example of this occurrence. Its rarity may be judged of by the fact that it is the only case that has occurred among 9442 tooth extractions performed in the Brunswick Hospital during 1859–66. A molar tooth was easily removed from the jaw of a tailor, 21 years of age, on account of caries. The bleeding, without being great, persisted in spite of astringents, and it was then stated that he, as well as his father and brother, were subjects of hemorrhagic diathesis. In the course of the night, severe bleeding came on, and he was brought to the hospital in an anæmic state, being scarcely conscious and his pulse hardly perceptible. The bleeding still continued, but was at last arrested by a conical cork plug. He was sufficiently recovered at the end of four days to leave the hospital, but having removed the plug the next day, profuse bleeding came on again, and it could only be arrested after several applications of the actual cautery. His strength was reduced to the lowest ebb, but by the aid of stimuli he was rallied. At the end of three days, in spite of all warning, he again removed the plug, and the bleeding again recurred, and was arrested at the end of several hours by plugging and cautery. However, the patient's strength was too far gone to rally this time, and he died on the day week that the tooth had been extracted The autopsy threw no light on the cause of the bleeding.—*Med. Times and Gaz., from Virchow's Archiv.*

